# Increased *N*-glycosylation of Asn^88^ in serum pancreatic ribonuclease 1 is a novel diagnostic marker for pancreatic cancer

**DOI:** 10.1038/srep06715

**Published:** 2014-10-22

**Authors:** Daisuke Nakata

**Affiliations:** 1 AIA Research Group, Department of Reagent Development, Division of Bioscience, Tosoh Corporation

## Abstract

Alterations of carbohydrate structures in cancer cells are the most promising targets for developing clinical diagnostic reagents. Pancreatic cancer is one of the most difficult cancers to diagnose because it lacks definitive symptoms. Two antibodies were raised against human pancreatic ribonuclease 1 that bind to the enzyme containing unglycosylated Asn^88^, but not when its Asn^88^ is *N*-glycosylated. Differential studies using these antibodies in immunoassays and Western blot analyses showed a significant increase in the serum levels of pancreatic ribonuclease 1 containing *N*-glycosylated Asn^88^ in pancreatic cancer patients compared with normal human subjects. Focusing on the increase in an *N*-glycosylated Asn residue of serum pancreatic ribonuclease 1, specifically Asn^88^, affords a new diagnostic marker for pancreatic cancer. This is the first report of a diagnostic cancer marker that takes advantage of the presence or absence of *N*-glycosylation at a specific Asn residue of a glycoprotein.

Alterations in glycan structures in cancers that arise from changes in the activity of glycosyltransferases or in the transcriptional regulation of their genes are considered to reflect the abnormal environment of cancer tissue^1^. Such structural changes have been used to develop a number of serum tumor markers, including CA19-9, sialyl lewis X, α-fetoprotein^2^, and are an important and promising area for developing new *in vitro* diagnostic (IVD) reagents. Studying changes in peripheral glycan structures in many cancers has been an active area to develop new biomarkers^2^,^3^. In contrast to global structural changes, there is no reported study of the presence or absence of *N*-glycan linked to a specific Asn residue within glycoproteins that correlate with diseases, with the exception of genetic disorders of glycosylation^4^,^5^. Although methods are available to detect *N*-glycosylated Asn residues^6^,^7^,^8^, it is difficult to use them to quantitatively determine the *N*-glycosylation status of specific targeted glycoproteins in human sera samples.

Pancreatic cancer (PaCa) is one of the most difficult cancers to diagnose because of the absence of early specific symptoms, and the lack of suitable diagnostic tests. The structural changes in the *N*-glycans of pancreatic ribonuclease 1 (RNase1) present in PaCa cells or patients' sera may serve as a diagnostic marker for PaCa^9^,^10^,^11^. RNase1 is secreted and has three consensus *N*-glycosylation motifs (sequon). Incomplete *N*-glycosylation of RNase1 in healthy pancreatic tissue, PaCa cells, and in the sera of PaCa patients' manifests as heterogeneity of its molecular mass^10^,^11^,^12^. However, the alterations of the presence of *N*-glycosylation in serum RNase1 have never been reported previously with respect to PaCa.

In this study, the development of anti-RNase1 antibodies that specifically recognize an unglycosylated Asn^88^ within RNase1 only when not *N*-glycosylated is reported. Differential analyses using these specific antibodies with other antibodies, which recognize all RNase1 molecules irrespective of the state of *N*-glycosylation, allowed for the quantitative determination of all RNase1 molecules and RNase1 with unglycosylated Asn^88^. The difference between the total level of RNase1 and the level of RNase1 with unglycosylated Asn^88^ thus provides a new method demonstrating that the level of RNase1 with *N*-glycosylation at Asn^88^ was significantly increased in the sera of PaCa patients, providing a new diagnostic marker for PaCa. This is the first report describing a glycan cancer marker based on the novel concept of detecting whether a specific Asn residue within a sequon (*N*-glycosylatable Asn) is *N*-glycosylated or not.

## Results

### An anti-RNase1 antibody with a characteristic reactivity with *N*-glycosylation site-deficient mutants of RNase1

**Figure 1:**
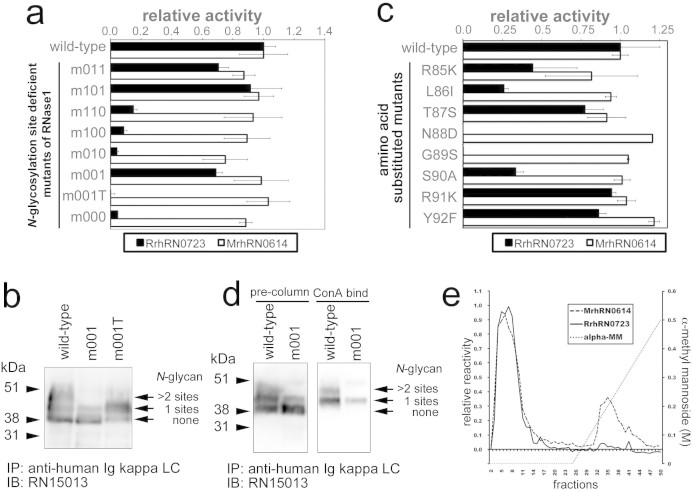
Characterization of an anti-human RNase1 antibody, RrhRN0723. The RrhRN0723 mAb was characterized using the *N*-glycosylation site-deficient or amino acid residue substitution mutants of RNase1 described in Methods. In panel a, the reactivity of the RrhRN0723 and MrhRN0614 mAbs against eight *N*-glycosylation site-deficient mutant and a wild-type protein was determined. Panel b shows the Western blot analyses of the wild-type, m001, and m001T proteins. Both m001 and m001T were partially *N*-glycosylated as indicated by the doublet band. In panel c, the single amino acid-substitution mutants from Arg^85^ to Tyr^92^ were subjected to the ELISAs to determine the minimal epitope recognized by the RrhRN0723 mAb. The essential residues were determined if the substitution of amino acids reduced the reactivity of the RrhRN0723 mAb to <50%. In panels a and c, the black and white bars represent the relative reactivity of each mutant with the RrhRN0723 and MrhRN0614 mAbs compared with wild-type, respectively. In panels *d* and *e*, the m001 mutant protein was subjected to ConA affinity chromatography to enrich the glycosylated form, and the eluted proteins was analyzed using Western blotting (*d*) and ELISAs (*e*) with the RrhRN0723 and MrhRN0614 mAbs. In panel *d*, Western blot analysis of wild-type and the m001 mutant are shown. *N*-glycosylated m001 (bound to ConA) was purified from partially glycosylated m001 mutant protein expressed in CHO-K1 cells (shown as pre-column m001) using the ConA column. In panel *e*, the m001 protein eluted from the ConA column was probed with the MrhRN0614 (dashed line) and RrhRN0723 (solid line) antibodies. The dotted line indicates the linear gradient of α-methyl mannoside. The blots in panel *b* and *d* were performed on the same blot membranes and shown as cropped images, respectively. In panel *a* and *c*, the bars and error bars represent the average and standard deviation values, respectively. Every experiment was performed for multiple times to confirm the reproducibility of experiments.

The *N*-glycan of RNase1 in serum from patients with PaCa patients is characterized by excess sialylation^10^ and core-fucosylation^11^ compared with that of healthy donors. To generate a monoclonal antibody (mAb) specific for the *N*-glycosylation state of RNase1, antibodies were raised in rodents against human RNase1 and subjected to selection using a series of *N*-glycosylation site-deficient mutants of RNase1. Recombinant mutant proteins that were partially or entirely deficient in *N*-glycosylation were generated by the substitution of Ala for the third amino acid (Ser or Thr) within the sequon (Table 1, Supplementary Fig. 1). Among the antibodies tested using the ELISA described in Methods, an antibody designated RrhRN0723 failed to detect the mutant proteins designated m110, m010, m100, and m000 (Fig. 1a). In contrast, an antibody designated MrhRN0614 reacted with all mutants as well as with the wild-type RNase1. The shared mutation among the mutants was the substitution of Ser^90^ by Ala in the third *N*-glycosylation site that prevented *N-*glycosylation of Asn^88^. There are three possible explanations for the lacks of binding activity of RrhRN0723 mAb to these mutants. First, RrhRN0723 mAb binds to only *N*-glycan moiety on Asn^88^. Second, peptide-backbone including Ser^90^ residue is an essential for binding of RrhRN0723 mAb and the *N*-glycan on Asn^88^ is not involved in at all. Third, both Ser^90^ residue and *N*-glycan moiety on Asn^88^ is involved in the binding of RrhRN0723 mAb.

### Antibody RrhRN0723 recognizes a six amino acid peptide moiety of RNase1 that includes Asn^88^

To determine the role of *N*-linked glycan on Asn^88^ of RNase1 in the reactivity of the RrhRN0723 mAb, a mutant with Thr^90^ substituted for Ser^90^ (m001T) was generated from m001 and analyzed as described in Methods. Although the m001T mutant has the third *N*-glycosylation site with Asn^88^ as does m001, both the recombinant protein of m001T with and without *N*-glycan linked to Asn^88^ were expressed in CHO-K1 cells (Fig. 1b). The substitution of Ser^90^ by Thr^90^ abolished reactivity with the RrhRN0723 mAb (Fig. 1a), suggesting that this antibody recognizes the Ser^90^ residue as part of its binding epitope, and does not recognize only the *N*-glycan moiety linked to Asn^88^. However, the possibility that the RrhRN0723 mAb simultaneously recognized both the *N*-glycan and the peptide chain could not be ruled out. Further analyses revealed, however, that substitution of each amino acid residue from positions 85 to 90 impaired the binding of the RrhRN0723 mAb to <50% compared with the wild-type protein, except for the substitution of Thr^87^ by the Ser residue (Fig. 1c). Collectively, these findings indicate that the six amino acid peptide moiety of RNase1, Arg**^85^**-Leu-Thr-Asn-Gly-Ser**^90^**, represents the minimal epitope essential for the reactivity of the RrhRN0723 mAb (Fig. 1c).

### The presence of the *N*-glycan linked to Asn^88^ inhibits the binding of the RrhRN0723 mAb to RNase1

Because the *N*-glycosylatable Asn^88^ is located in the center of the binding epitope, the possibility that the presence of *N*-glycan linked to Asn^88^ may affect the binding of the RrhRN0723 mAb was further investigated. For this purpose, the m001 mutant with only one *N*-glycan chain linked to Asn^88^ was used. The m001 mutant protein expressed in CHO-K1 cells, which was partially *N*-glycosylated at Asn^88^, was subjected to concanavalin A (ConA) affinity chromatography to separate the *N*-glycosylated from the unglycosylated form (Fig. 1d left panel). The fractions of ConA column chromatography were analyzed using the ELISA with the RrhRN0723 and MrhRN0614 mAbs (Fig. 1e). *N*-glycosylated m001 in the ConA-bind fraction (Fig. 1d right panel) was not detected by the RrhRN0723 mAb, in contrast to the MrhRN0614 mAb; although, unglycosylated m001 was detected by both antibodies (Fig. 1e). These results show that the recognition of the six amino acid peptide sequence by the RrhRN0723 mAb was inhibited by the presence of the *N*-glycan chain linked to Asn^88^.

### Analysis of serum RNase1 levels in patients with PaCa using the RrhRN0723 antibody

**Figure 2:**
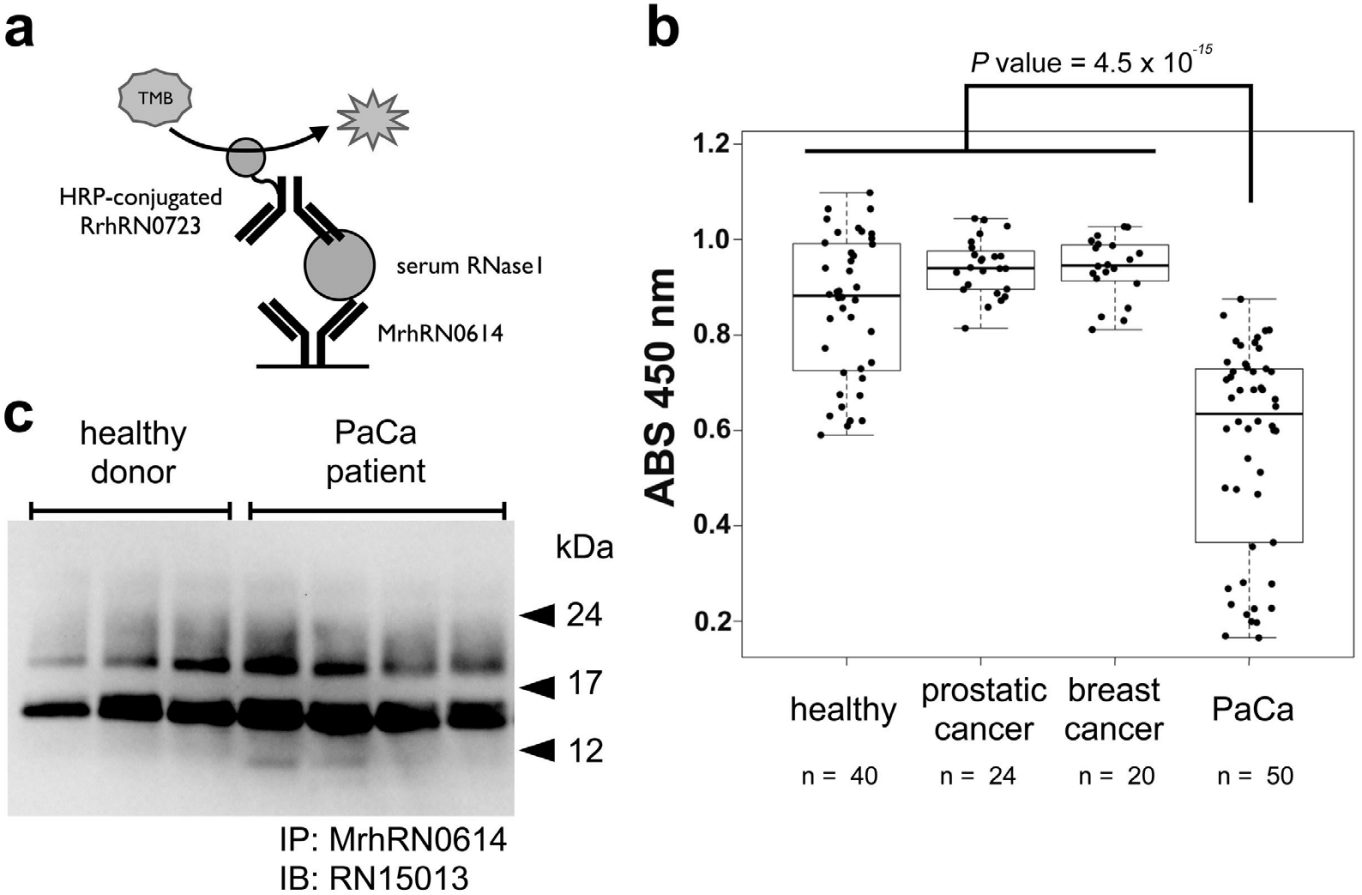
Sandwich ELISA and Western blot analyses of serum RNase1. A diagrammatic summary of the sandwich ELISA analyses is shown in panel *a*. MrhRN0614 and HRP-conjugated RrhRN0723 antibodies were used for capture and detection antibodies, respectively. Panel *b* shows the levels of serum RNase1 in healthy donors and patients with several types of cancer, determined using a sandwich ELISA with MrhRN0614 and RrhRN0723 mAbs. Statistical analysis of serum RNase1 detection of patients with PaCa or other cancers were performed using R statistic software and the *P* value was calculated using a Wilcoxon-test^22^,^23^. The boxes are indicating interquartile ranges for each group of specimens. The length of the error bars are 1.5 fold length of the interquartile range. Panel *c* shows the results of the Western blot analyses of serum from healthy donors and patients with PaCa patients using the MrhRN0614 antibody to immunoprecipitate RNase1. Multiple bands were detected due to the heterogeneity of *N-*glycosylation. The result of a single blot membrane was shown in panel *c* as a cropped image.

The levels of RNase1 in sera from healthy donors, patients with PaCa, and patients with other diseases were determined using an ELISA with the MrhRN0614 and RrhRN0723 mAbs (Fig. 2a). The serum level of RNase1 was significantly decreased in patients with PaCa compared with healthy donors and patients with other diseases (Fig. 2b, *P* = 2.2 × 10^−16^). Western blot analyses of serum RNase1 after immunoprecipitation using the MrhRN0614 mAb indicated that the serum RNase1 levels in patients with PaCa were not significantly different compared with those of healthy donors (Fig. 2c). These data indicate that the amount of RNase1 without *N*-glycosylation of Asn^88^ detected by the RrhRN0723 mAb was significantly decreased in the sera of patients with PaCa, although there was no significant difference in the total amount of serum RNase1 of the tested specimens. These results address the hypothesis that the *N*-glycosylation at Asn^88^ of serum RNase1 is increased in patients with PaCa.

### Development of an assay to specifically detect unglycosylated Asn^88^ in denatured RNase1

**Figure 3:**
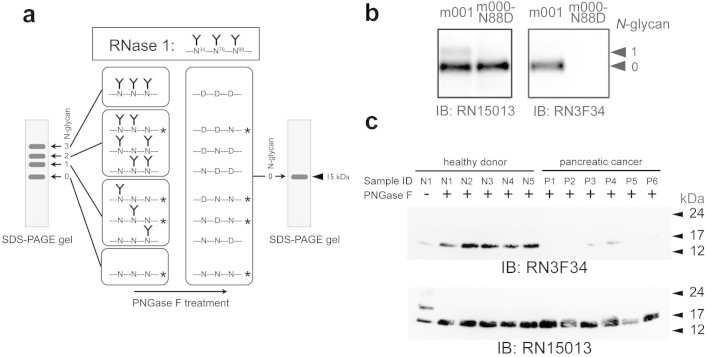
Western blot analysis using the RN3F34 antibody to determine the presence of unglycosylated Asn^88^ of serum RNase1 from healthy donors and patients with pancreatic cancer. Panel *a* shows a diagram of the experimental design of the Western blot analysis combined with PNGase F treatment to detect Asn^88^-free RNase1 in serum specimens. The upper panel shows a simplified model of fully glycosylated RNase1. Untreated RNase1 migrates as four bands on the SDS-PAGE gel according to the degree of *N*-glycosylation. After PNGase F treatment, all forms of RNase1 will migrate at 15 kDa. The asterisked RNase1, which originally lacked an *N*-glycan chain linked to Asn^88^, is detected by RN3F34. Panel *b* shows the Western blot analyses, which indicates the differences between the reactivities of RN3F34 with RNase1 mutants, m001 and m000-N88D. RN3F34 mAb did not detect the RNase1 mutant with an Asp substituted for Asn^88^. Panel *c* shows a representative Western blot of serum RNase1 from healthy donors and patients with PaCa. The detection of Asn^88^-free RNase1 by RN3F34 mAb is shown in the upper panel and the detection of total RNase1 with RN15013 mAb is shown in the lower panel. The blots in panel *b* and *c* were performed under the same experimental condition except for blotting with the different antibodies, respectively. These blots were shown as cropped images.

To more precisely determine the state of *N*-glycosylation at Asn^88^ of RNase1 in the sera of patients with PaCa, a Western blot analysis was developed (Fig. 3a). Peptide-*N*-gylcosidase F (PNGase F) deamidates *N*-glycosylated Asn to produce Asp residues by cleaving an *N*-glycan from a glycoprotein^13^. Serum RNase1 migrates as four distinct bands in an SDS-PAGE gel according to its degree of *N*-glycosylation. After deglycosylation by PNGase F, the formerly *N*-glycosylated RNase1 isoforms (top three bands Fig. 3a, left panel) migrate as a single band with an approximate molecular mass of 15 kDa. The anti-RNase1 mAb RN15013, which recognizes the *N*-terminal peptide sequence, detects each deglycosylated peptide as a single 15 kDa band. In contrast, an antibody that binds to the peptide moiety by recognizing RNase1 Asn^88^, but does not bind to the isoform with the Asp^88^ residue, detects only the RNase1 that originally had an unglycosylated Asn^88^ in the 15 kDa band (asterisked molecules in Fig. 3a). Therefore, the total RNase1 (differentially glycosylated) and RNase1 with unglycosylated Asn^88^, named hereafter “Asn^88^-free RNase1,” can be distinguished on SDS-PAGE gels following PNGase F treatment. The RrhRN0723 mAb did not detect any of the denatured forms of RNase1. Another mAb, RN3F34, was raised that recognized the same peptide epitope of RNase1 as RrhRN0723 (see Methods). The RN3F34 mAb did not detect the upper band of the m001 mutant, which was detected by the RhRN15013 mAb (Fig. 3b), indicating that the RN3F34 mAb did not react with RNase1 that was *N*-glycosylated on Asn^88^. Further, RN3F34 did not detect the mutant m000-N88D (Asn^88^ replaced with Asp, see Table 1 and Supplementary Fig. 1). Therefore, using RN3F34 combined with PNGase F treatment, it was possible to detect Asn^88^-free RNase1 in serum.

### Qualitative analyses of Asn^88^-free RNase1 in sera of PaCa patients

Qualitative analyses of Asn^88^-free RNase1 in sera of healthy donors and patients with PaCa were performed using RN3F34 and RhRN15013 after immunoprecipitation with the MrhRN0614 mAb. There was no significant difference in the level of total RNase1 detected by the RhRN15013 mAb between healthy donors and patients with PaCa (Fig. 3c). However, the level of serum RNase1 detected by the RN3F34 mAb in patients with PaCa was significantly less than in healthy donors (Fig. 3c upper panel). These results show that the level of Asn^88^-free RNase1 was significantly decreased in sera from patients with PaCa, supporting the hypothesis that *N*-glycosylation at Asn^88^ of serum RNase1 was significantly increased in the patients with PaCa.

### Enzyme immunoassay (EIA) of Asn^88^-free and total RNase1 for diagnosis of PaCa

**Figure 4:**
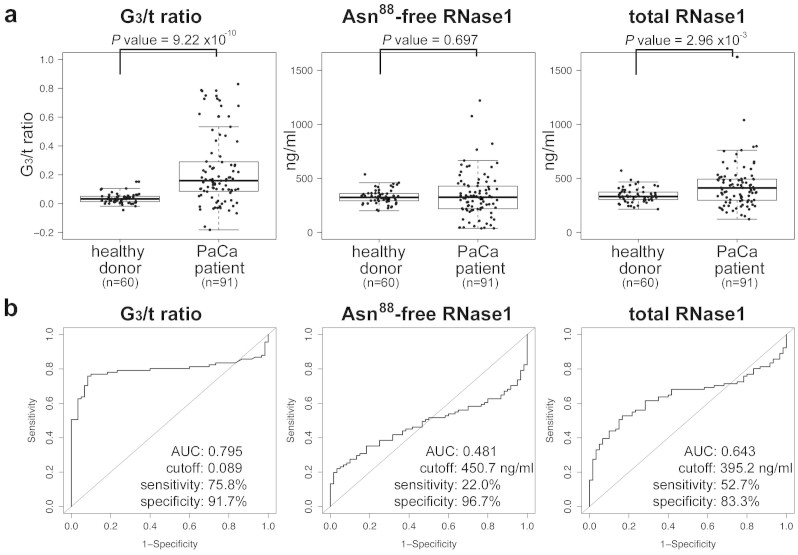
Enzyme Immunoassay and calculated G_3_/t ratio of serum RNase1 of healthy donors and patients with PaCa. In panel *a*, the box-plot overlaid with the scatter plot shows the distributions of G_3_/t ratio, Asn^88^-free RNase1 and total RNase1 concentrations in sera of healthy donors (*n* = 60) and PaCa patients (*n* = 91). The measurements of Asn^88^-free RNase1 and total RNase1 concentrations were independently performed twice with duplicate assays. The coefficient of variation (CV) of over 95% assays was within 5%, and the averages of CV were 1.7% and 2.4% for Asn^88^-free and total RNase1, respectively. The boxes are indicating interquartile ranges for each group of specimens. The length of the error bars are 1.5 fold length of the interquartile range. The values of the median and interquartile range of each analysis are summarized in Table 2. *P* values of each analysis comparing healthy donors and patients with PaCa were determined using the Wilcoxon-test component of the R statistical software suite. In panel *b*, the results of Receiver Operating Characteristic analyses using R statistical software with the pROC suite for ROC analysis^22^,^24^ are shown for each analysis. The values of area under curve (AUC), cut-off, sensitivity and specificity are shown in each ROC plot. The sample size was confirmed to be appropriate for these comparative analyses with a power of 0.80.

The results of EIA analyses for Asn^88^-free RNase1 using the RrhRN0723 mAb did not reliably distinguish patients with PaCa from healthy donors (Fig. 4a center panel, *P* = 0.594) due to variation in the concentration of total serum RNase1 concentrations. For more accurate analyses of *N*-glycosylation at Asn^88^ of serum RNase1, two EIA reagents were developed for use with an Automated Immunoassay Analyzer (Tosoh Corp., http://www.tosohbioscience.com), which is used worldwide in clinical laboratories and hospitals (see Methods). One EIA reagent was designed to determine the concentration of Asn^88^-free RNase1 using the RrhRN0723 mAb, and the other was designed to determine total RNase1 concentration using the RrhRN1111 mAb. The latter mAb bound all RNase1 isoforms irrespective of the state of *N*-glycosylation (Supplementary Fig. 2). The ratio of RNase1 with *N*-glycosylated Asn^88^ in the total population of RNase1 (RNase1 *N*-glycosylated at the 3rd (Asn^88^) site per total RNase1, G_3_/t ratio) was calculated as follows: 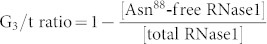


The G_3_/t ratio of the serum RNase1 of patients with PaCa was significantly higher compared with healthy donors, in contrast to the concentration of Asn^88^-free RNase1 or total RNase1. Receiver Operating Characteristic (ROC) curve analysis showed that the G_3_/t ratio had sufficient predictive power for the diagnosis of PaCa (Fig. 4b, area under curve value: 0.795, cutoff value: 0.079, sensitivity: 75.8% and specificity: 91.7%).

## Discussion

The present study shows that *N*-glycosylation at Asn^88^ in serum RNase1 was significantly increased in patients with PaCa. This finding was established using an immunoassay that was developed to determine the level of total RNase1 compared with that of RNase1 that was not *N-*glycosylated on Asn^88^ (Asn^88^-free). The increase levels of RNase1 with *N*-glycosylated Asn^88^ in the sera of patients suspected of having PaCa thus shows promise as a novel diagnostic marker for screening and detection of PaCa. The immunoassay and Western blot-base analysis were developed for detecting this change in *N*-glycosylation of RNase1 using RrhRN0723 mAb and RN3F34 mAb, respectively. Both antibodies directed against human RNase1 recognize the peptide region surrounding the Asn^88^ *N*-glycosylation site containing unglycosylated Asn^88^, but not when its Asn^88^ is *N*-glycosylated. The EIA analysis using RrhRN0723 which allows the high-throughput and quantitative detection of the levels of Asn^88^-free RNase1 is suitable for clinical purpose. In contrast, the Western blot analysis using RN3F34 which allows the qualitative detection of the changes in *N*-glycosylation at Asn^88^ is good for research tool rather than clinical purpose.

The results of the ELISA using this antibody to detect serum RNase1 revealed that the level of RNase1 recognized by the RrhRN0723 mAb was significantly decreased in the sera of patients with PaCa compared with healthy donors or patients with diseases other than PaCa (Fig. 2). There were some patients with PaCa, for example, whose levels of Asn^88^-free RNase1 were not distinguishable from those of healthy donors, resulting in an apparent decrease in the overall capability of the Asn^88^-free RNase1 assay (Fig. 4a). Further analyses revealed that the cause was an elevation in the level of total RNase1 in the sera of patients with PaCa (Fig. 4a). Studies conducted during the 1970s and 1980s showed that the serum levels or activity of RNase1 were elevated in patients with PaCa, leading to the conclusion that neither variable served as a specific marker of PaCa^14^,^15^,^16^,^17^. While the results of my present study is similar in some respects to those presented earlier (Fig. 4b, area under curve of the ROC = 0.643 for total RNase1). In contrast, the G_3_/t ratio described here to measure the *N*-glycan attached to Asn^88^ is importantly more accurate and unambiguous in distinguishing patients with PaCa from healthy donors (Fig. 4b).

The functional role of serum RNase1 in association with PaCa is poorly understood, although the elevation of total RNase1 in serum of PaCa patients has been known from 1970's^14^,^15^,^16^,^17^. It is also unclear whether the *N*-glycosylated RNase1 at Asn^88^ has differential features compared with RNase1 with unglycosylated Asn^88^, yet. Further analyses will be needed to elucidate the functional role of highly *N*-glycosylated RNase1 in the development and progression of PaCa.

This study has focused on the presence or absence of *N*-glycan linked to Asn^88^ in serum RNase1, in contrast to previous studies describing overall changes in the structure of *N*-glycan in PaCa cells^10^,^11^. Although the structural changes in the *N*-glycan may be explained by changes in the transcriptional regulation of genes encoding enzymes involved in glycosylation, there is no clear explanation as to why *N*-linked glycosylation of Asn^88^ in serum RNase1 is increased in PaCa patients. The presence or absence of *N*-glycan on Asn residues is determined to a large extent by the activity of oligosaccharyltransferase (OST) complexes. OST is a key regulatory enzyme in *N*-linked glycosylation that catalyzes the transfer a 14 sugar-oligosaccharide moiety from a dolichol-linked precursor to the growing nascent glycoproteins in the lumen of the endoplasmic reticulum (ER)^18^. Thus, the difference in the degree of *N*-glycosylation of Asn^34^, Asn^76^, and Asn^88^ in RNase1 may result from the differential activities of OST complexes in a single cell. Up-regulation of the levels of some OST complexes in PaCa cells may increase the level of glycosylation of RNase1 Asn^88^. Therefore, my future studies will investigate the comparative activities of OST complexes in PaCa and normal cells.

In contrast, preliminary results suggest that this alteration may be due to the structure of RNase1. Among the mutant forms of RNase1 generated to determine the binding epitopes of the RrhRN0723, RrhRN1111, and MrhRN0614, there were mutants that were not recognized by these antibodies. For example, ELISA and Western blot analyses of the His^119^, Asp^121^, and Ser^123^ deletion mutants are shown in Supplementary Fig. 4. In the ELISA analysis, none of these antibodies bound to the mutated RNase1 lacking His^119^ (Supplementary Fig. 4a). Western blot analyses show that the His^119^ deletion mutant was heavily *N*-glycosylated compared with wild-type and the other deletion mutants (Supplementary Fig. 4b).

Because the His^119^ residue is highly conserved among mammalian pancreatic ribonucleases and is involved in the active site of bovine RNaseA^19^, the deletion of His^119^ may possibly induce a conformational change. Thus, the MrhRN0614 mAb, which did not bind denatured RNase1, failed to detect the His^119^ deletion mutant in the ELISA. Moreover, full *N*-glycosylation of the His^119^ deletion mutants, as revealed by Western blot analyses, suggests that a possible conformational change in RNase1 may affect the *N*-glycosylation of Asn residues. Collectively, these findings are in accord with the hypothesis that a conformational alteration in RNase1 during *de novo* synthesis in the ER of PaCa cells may enhance the ability of OST complexes to transfer the glycan moiety to Asn^88^ of RNase1 compared with those of normal cells. The cause of the conformational changes during the *de novo* synthesis of RNase1 in PaCa cell is unknown, although, ER stress under hypoxic conditions may be one possibility^1^.

Antibodies that form part of the components of IVD reagents used to detect the cancer marker CA15-3 recognize a peptide moiety that includes an *O*-glycosylation site that is exposed by decreased *O*-glycosylation of MUC1 that is associated with breast cancer^20^. Serum transferrin of patients with congenital disorder of glycosylation is under-*N*-glycosylation and has been used to determine genotypes of deficiency of enzymes involved in glycan biosynthesis^5^. In these cases, the abnormal absence of the glycan chain is used for detection of different disease states. In contrast, the present study is the first to identify a cancer marker that distinguishes patients with PaCa by detecting altered levels of *N*-glycosylation of a specific amino acid residue, Asn^88^, in RNase1. This “*N*-glycosylation marker” serves as a cancer marker based on a novel concept of detecting whether a specific *N*-glycosylatable Asn is *N*-glycosylated or not.

To date, carbohydrate disease makers including sialyl lewis A (CA19-9), sialyl lewis X and core-fucosylation of alpha-fetoprotein are clinically utilized as IVD reagents. Other structural changes in glycans expressed in cancer cells identified by glycomics research have shown promise as cancer markers^2^,^3^. However, there are few examples of exploiting a structural change of an *N*-glycan revealed by glycomics research that serve as the basis of an immunoassay, which is the most frequently used format for clinical IVD reagents^21^. A possible explanation is the low immunogenicity of glycan chains. In contrast to the conventional glycan markers using anti-carbohydrate antibodies, the advantage of an *N*-glycosylation marker is that antibody binding requires recognition of the peptide moiety as described herein. Thus, such an antibody will bind to its target with sufficient affinity and specificity for use in an IVD assay compared with antibodies against only the carbohydrate moieties.

Although further clinical research is required to evaluate the suitability for clinical application of the marker for RNase1 described herein, it is likely that it will contribute to the diagnosis and cure of patients with PaCa. In the clinical evaluation researches of this marker started in Tosoh Corporation, it will be particularly important to determine if this type of change in glycosylation is a late or early event in tumor progression as well as its correlation with the clinicopathological factor of patients. The findings reported here should stimulate research to identify similar new markers that may serve to improve the diagnosis and therapy of other diseases.

## Methods

### Materials

All synthetic peptides, goat anti human immunoglobulin kappa light chain (hIgk-LC) antibody, recombinant Protein-L and the other chemicals were purchased from Sigma-Aldrich. The MGC cDNA clone of human pancreatic RNase1 was purchased from Thermo Fisher Scientific Inc. The PaCa cell line Capan-1 (ATCC number HTB-79), was obtained from the American Type Culture Collection. Female mice (Balb/c strain) and rats (WKY/NcrlCrlj strain) were purchased from Charles River Laboratories Japan, Inc.

### Serum samples

Serum samples of patients and healthy donors were obtained from several venders who collect samples with informed consent under an IRB-approved protocol. Specimens were voluntarily provided by the employees of Tosoh Corporation with informed consent. The Ethics Committee of the Bioscience Division of Tosoh Corporation approved the experiments using human specimens. The serum specimens from patients clinically diagnosed with PaCa were obtained from BioTheme Inc. (Davie, Florida) Nova Biologics, Inc. (Oceanside, California) and ProMedDx Inc. (Norton, Massachusetts). The serum specimens from healthy donors, patients with clinically diagnosed breast and prostatic cancer were obtained from ILSbio, LLC. (Chestertown, Maryland) and ProMedDx Inc.

### Development of anti-human RNase1 antibodies

All experiments using living vertebrates to generate antibodies were performed in accordance with the relevant guideline of Japanese government. Monoclonal antibodies that recognized denatured or intact forms of human pancreatic RNase1 were generated by immunizing mice or rats with synthetic peptides or recombinant proteins, respectively. The hybridoma cell that expresses the antibody RhRN15013, which binds the *N-*terminal peptide of denatured RNase1, was generated from B-cells of the retroperitoneal and inguinal lymph nodes of rats immunized with a synthetic peptide corresponding to the *N*-terminal 14 amino acid residues of RNase1 (KESRKKFQRQHMDS). This peptide was conjugated to KLH (Thermo Fisher Scientific Inc.) followed by selection using the same peptide conjugated to biotin at its *C*-terminus. The hybridoma expressing RN3F34, which binds to the *N-*glycosylation site including Asn^88^ of denatured RNase1, was generated using the same procedures described, except that the animals were immunized with the synthetic peptide (acetyl-RLTNGSRYPNC) conjugated to KLH corresponding to Arg^85^ to Asn^94^ of RNase1 and the SRLTNGSRYPNC and SRLTDGSRYPNC conjugated to BSA for selection of hybridoma cells. The hybridoma cells were selected on the basis of those that produced the desired antibodies that reacted with the intact sequence and not with the peptide in which Asn^88^ was substituted with Asp. The antibodies which recognize the intact forms of RNase1 were generated using the same procedures as described above, except using spleen B-cells of mice for the MrhRN0614 antibody and B-cells of retroperitoneal and inguinal lymph nodes of rats that were immunized with recombinant proteins to raise RrhRN0723 and RrhRN1111 antibodies. To obtain the recombinant proteins, the insect cell Sf9 was transfected with the plasmid vector pIZ-KFHhRNase1, which was constructed by inserting the entire hIgk-LC linked to the FLAG and 6× His tags upstream of the RNase1 gene of the insect expression vector pIZ-v5His (Life Technologies). The protein secreted into the medium of transfected Sf9 cells was purified using a protein-L affinity chromatography. For selecting hybridoma cells expressing the MrhRN0614 antibody, the purified recombinant protein described above was used in ELISAs. For selecting hybridoma cells expressing the RrhRN0723 and RrhRN1111 antibodies, the intact form of RNase1 purified from the conditioned medium of Capan-1 cells and captured by the MrhRN0614 antibody on the ELISA plate was used.

### Molecular cloning and expression of wild-type and mutated recombinant RNase1 molecules

Expression vectors for wild-type and mutated recombinant human RNase1 were constructed and used to transfect CHO-K1 cells as described below. The gene encoding human RNase1, including *N-*terminal tags, was amplified using PCR from pIZ-KFHhRNase1 followed by insertion into the pcDNA3.1-mycHis vector (Life Technologies) to construct a mammalian expression vector to express human RNase1 (pcDNA-KFHhRNase1). For the construction of the substitution mutants, mutagenesis of pcDNA-KFHhRNase1 plasmid was performed according to the protocol supplied by the manufacturer for the Prime Stat PCR mutagenesis Kit (Takara Bio Inc., Japan). The recombinant proteins were expressed in CHO-K1 cells transiently transfected with wild-type and mutated plasmids and analyzed using the ELISA or immunoprecipitated with anti-hIgk-LC antibody followed by the Western blot analyses with RhRN15013.

### ELISA analyses to identify the epitope recognized by the anti-RNase1 antibody

The ELISA immunoassay to characterize the anti-RNase1 antibody was carried out as follows: Five hundred nanograms of anti-hIgk-LC antibody in 50 μl of 20 mM phosphate buffer (pH 7.2) containing 150 mM NaCl (PBS) was added to the 96-well immunoassay plate and kept at 4°C overnight. The wells were washed with 20 mM Tris-HCl buffer (pH 7.2) containing 150 mM NaCl (TBS) followed by blocking with 3% BSA in TBS for 2 h. After removing the blocking solution, 50 μl of the supernatant of CHO-K1 cells transfected with wild-type and mutated pcDNA-KFHhRNase1 plasmids was added to each well and kept at room temperature for 1 h. After washing with TBS containing 0.05% Tween-20 (TBST), 25 ng of HRP-conjugated RrhRN0723, RrhRN1111, or MrhRN0614 antibodies in TBST containing 1% BSA was added to the well. After incubation at room temperature for 60 min followed by washing with TBST, the amount of each HRP-conjugated antibody bound to the plate was measured using TMB solution.

### ConA-affinity purification of recombinant RNase1

The culture medium of CHO-K1 cells expressing the recombinant KFH-RNase1 m001 mutant was subjected to chromatography using a ConA-conjugated HiTrap column (GE Healthcare UK Ltd.) to separate *N*-glycosylated from unglycosylated forms using an HPLC system. After washing the column, the *N*-glycosylated m001 mutant was eluted using a linear gradient of α-methyl mannoside (0–500 mM). Fraction (1 ml) were subjected to the ELISA described above.

### ELISA of pancreatic RNase1 in human serum

The ELISA was carried out as follows: Twenty five nanograms of the MrhRN0614 mAb in 50 μl PBS was added to a 96-well immunoassay plate and kept at 4°C overnight. The wells were washed with TBS followed by blocking with 3% BSA in TBS for 2 h. After removing the blocking reagents, diluted human serum was added to each well and kept at room temperature for 1 h. After washing with TBS containing 0.05% Tween-20 (TBST), 25 ng of HRP conjugated RrhRN0723 mAb in TBST containing 1% BSA was added to the wells and keep at room temperature for 1 h. After washing with TBST, the amount of HRP-RrhRN0723 mAb bound to the plate was measured using TMB solution. For the calculation of the amount of RNase1, the RNase1 in the culture medium of Capan-1 cell was purified using an RrhRN0723 antibody-conjugated HiTrap column, followed by the determination of protein amount using the BCA protein assay kit (Thermo Fisher Scientific Inc.). On each immunoassay plate, serially diluted purified RNase1 (0–100 ng/ml) was measured as a standard.

### Immunoprecipitation and Western blot analysis of RNase1 from human serum

Immunoprecipitation was performed using the MrhRN0614 mAb adsorbed to Dynabead-anti mouse IgG beads (Life Technologies). Ten microliters of human serum was diluted with 90 μl of PBST followed by mixing with an appropriate amount of MrhRN0614 antibody-beads for 1 h. The beads washed with PBST three times were suspended in 7 μl of PNGase F denaturing buffer and incubated at 37°C for 1 h. By adding 1 μl of each NP-40 solution, reaction buffer, and PNGase F (New England Biolabs Inc.), then incubating at 37°C for 1 h, the precipitated RNase1 was released from beads and deglycosylated. The reaction mixtures were divided in two and separated using SDS-PAGE. The PVDF membranes transferred from SDS-PAGE gels were probed with biotin-labeled RhRN15013 and RN3F34 mAb, respectively.

### EIA detection of serum RNase1

To measure the levels of Asn^88^-free and total RNase1 in serum, two EIA reagents for use with an Automated Immunoassay Analyzer (AIA, Tosoh Corporation, Japan), were developed. The AIA and its test cup are shown in Supplementary Fig. 3. Briefly, for total RNase1 detection, a new anti-RNase1 antibody, RrhRN1111, which bound serum RNase1 independently of the state of *N*-glycosylation, was used. To prepare EIA reagents for Asn^88^-free RNase1 or for total RNase1 measurements, the test cups for the AIA reagent containing the MrhRN0614 mAb (720 ng per test cup) adsorbed to the magnetic carrier beads were added the alkaline phosphatase-conjugated RrhRN0723 mAb (50 ng per test cup) or to the RrhRN1111 mAb (50 ng per test cup) in 100 μl of TBS containing 1% BSA followed by freeze-drying under vacuum. For the EIA measurements, the serum specimens were diluted with 20 volumes of TBST containing 1% BSA. Using two EIA reagents with the AIA-2000 analyzer, the amount of Asn^88^-free and total RNase1 in the sera of healthy donors and patients with PaCa were determined. The concentrations of each analyte were determined according to the calibration curves of serially-diluted RNase1 (0.0–100 ng/ml for Asn^88^-free RNase1, 0.0–106.5 ng/ml for total RNase1) expressed and purified from CHO-K1 cells, and the concentration was determined using authentic Asn^88^-free RNase1. The calibration curves for both analyses are shown in Supplementary Fig. 5. Statistical analyses were carried out using R statistic software^22^ with the pROC suite for ROC analysis^23^.

### Materials availability

The antibodies, MrhRN0614, RrhRN0723, RrhRN1111 and RN3F34 in this article are available for research-only purposes from Tosoh Corporation with the restrictive Material Transfer Agreement. The AIA reagents for detecting Asn^88^-free and total RNase1 will be available for research-only purposes from Tosoh Corporation.

## Author Contributions

D.N. designed and performed all experiments, analyzed data, and wrote the manuscript.

## Supplementary Material

Supplementary Information Supplementary Figures

## Figures and Tables

**Table 1:** *N*-glycosylation site-deficient RNase1 mutants

	amino acid sequence of originally *N*-glycosylation site in wild-type			
mutants	*1st (34*–*36)*	*2nd (76*–*78)*	*3rd (88*–*90)*	number of *N*-glycosylation site
wild-type	NMT	NSS	NGS	3
m011	NMA	NSS	NGS	2
m101	NMT	NSA	NGS	2
m110	NMT	NSS	NGA	2
m100	NMT	NSA	NGA	1
m010	NMA	NSS	NGA	1
m001	NMA	NSA	NGS	1
m001T	NMA	NSA	NGT	1
m000	NMA	NSA	NGA	0
m000-N88D	NMA	NSA	DGS	0

The three amino acid-residue consensus sequences for the three *N*-glycosylation sites in the wild-type enzyme and the possible number of *N*-glycosylation sites are shown for wild-type and mutant proteins.

**Table 2:** Statistical analyses of EIA measurements of serum RNase1 levels of healthy donors and patients with PaCa

specimens	sample size	G_3_/t ratio	Asn^88^-free RNase1	total RNase1
		*ratio*#	*ng/ml*#	*ng/ml*#
healthy donor	60	0.033 (0.014–0.051)	324.0 (293.5–359.9)	332.1 (304.8–373.2)
PaCa patient	91	0.159 (0.086–0.290)	325.7 (219.3–342.0)	411.3 (297.7–493.0)
			*ROC analysis*	
AUC		0.795	0.481	0.643
cutoff		0.089	450.7 ng/ml	395.2 ng/ml
sensitivity		75.8%	22.0%	52.7%
specificity		91.7%	96.7%	83.3%

^#^median (interquartile range) The statistical values are from Fig. 4.

## References

[b1] Kannagi R. *et al.* Altered expression of glycan genes in cancers induced by epigenetic silencing and tumor hypoxia: clues in the ongoing search for new tumor markers. Cancer Sci. 101, 586–593 (2010). 20085584 10.1111/j.1349-7006.2009.01455.x PMC11158919

[b2] Peracaula R. *et al.* Altered glycosylation in tumours focused to cancer diagnosis. Dis Markers. 25, 207–218 (2008). 19126965 10.1155/2008/797629 PMC3827805

[b3] An H. J., Kronewitter S. R., de Leoz M. L. A. & Lebrilla C. B. Glycomics and disease markers. Curr Opin Chem Biol. 13, 601–607 (2009). 19775929 10.1016/j.cbpa.2009.08.015 PMC2788081

[b4] Durand G. & Seta N. Protein glycosylation and diseases: blood and urinary oligosaccharides as markers for diagnosis and therapeutic monitoring. Clin Chem. 46, 795–805 (2000). 10839767

[b5] Grünewald S., Matthijs G. & Jaeken J. Congenital disorders of glycosylation: a review. Pediatr Res. 52, 618–624 (2002). 12409504 10.1203/00006450-200211000-00003

[b6] Kaji H. *et al.* Lectin affinity capture, isotope-coded tagging and mass spectrometry to identify *N*-linked glycoproteins. Nat Biotechnol. 21, 667–672 (2003). 12754521 10.1038/nbt829

[b7] Hägglund P., Bunkenborg J., Elortza F., Jensen O. N. & Roepstorff P. A new strategy for identification of *N*-glycosylated proteins and unambiguous assignment of their glycosylation sites using HILIC enrichment and partial deglycosylation. J. Proteome Res. 3, 556–566 (2004). 15253437 10.1021/pr034112b

[b8] Kaji H., Yamauchi Y., Takahashi N. & Isobe T. Mass spectrometric identification of *N*-linked glycopeptides using lectin-mediated affinity capture and glycosylation site-specific stable isotope tagging. Nat Protoc. 1, 3019–3027 (2006). 17406563 10.1038/nprot.2006.444

[b9] Fernandez-Salas E., Peracaula R., Frazier M. L. & de Llorens R. Ribonucleases expressed by human pancreatic adenocarcinoma cell lines. Eur. J. Biochem. 267, 1484–1494 (2000). 10691987 10.1046/j.1432-1327.2000.01148.x

[b10] Peracaula R. *et al.* Glycosylation of human pancreatic ribonuclease: differences between normal and tumor states. Glycobiology 13, 227–244 (2003). 12626415 10.1093/glycob/cwg019

[b11] Barrabés S. *et al.* Glycosylation of serum ribonuclease 1 indicates a major endothelial origin and reveals an increase in core fucosylation in pancreatic cancer. Glycobiology 17, 388–400 (2007). 17229815 10.1093/glycob/cwm002

[b12] Ribó M. *et al.* Heterogeneity in the glycosylation pattern of human pancreatic ribonuclease. Biol Chem Hoppe Seyler. 375, 357–63 (1994). 8074810

[b13] Plummer T. H. Jr, Elder J. H., Alexander S., Phelan A. W. & Tarentino A. L. Demonstration of peptide: *N*-Glycosidase F activity in Endo-beta-*N*-acetylglucosaminidase F Preparations. J Biol Chem. 259, 10700–10704 (1984). 6206060

[b14] Reddi K. K. & Holland J. F. Elevated serum ribonuclease in patients with pancreatic cancer. Proc Natl Acad Sci USA. 73, 2308–2310 (1976). 1065880 10.1073/pnas.73.7.2308 PMC430542

[b15] Doran G., Allen-Mersh T. G. & Reynolds K. W. Ribonuclease as a tumour marker for pancreatic carcinoma. J Clin Pathol. 33, 1212–1213 (1980). 7451669 10.1136/jcp.33.12.1212 PMC1146378

[b16] Weickmann J. L., Olson E. M. & Glitz D. G. Immunological assay of pancreatic ribonuclease in serum as an indicator of pancreatic cancer. Cancer Res. 44, 1682–1687 (1984). 6704974

[b17] Kurihara M. *et al.* Radioimmunoassay for human pancreatic ribonuclease and measurement of serum immunoreactive pancreatic ribonuclease in patients with malignant tumors. Cancer Res. 44, 2240–2243 (1984). 6713412

[b18] Stanley P., Schachter H. & Taniguchi N. *N*-*glycans*. In: Varki, A. *et al.*, *editors.* Essentials of Glycobiology. 2nd edition. Cold Spring Harbor Laboratory Press, Cold Spring Harbor, New York, 2009.

[b19] Crestfield A. M., Stein W. H. & Moore S. Alkylation and identification of the histidine residues at the active site of ribonuclease. J Biol Chem. 238, 2413–2420 (1963). 14023809

[b20] Price M. R. *et al.* Summary report on the ISOBM TD-4 Workshop: analysis of 56 monoclonal antibodies against the MUC1 mucin. San Diego, Calif., November 17–23, 1996. Tumour biol 19, 1–20 (1998). 9422084 10.1159/000056500

[b21] Kuno A. *et al.* A serum “sweet-doughnut” protein facilitates fibrosis evaluation and therapy assessment in patients with viral hepatitis. Sci Rep. 3, 10.1038/srep01065 (2013). 10.1038/srep01065 PMC3545220 23323209

[b22] Ihaka R. & Gentleman R. R: a language for data analysis and graphics. J Comp Graph Stat. 5, 299–314 (1996).

[b23] Bauer D. F. Constructing confidence sets using rank statistics. J Amer Statist Assoc. 67, 687–690 (1972).

[b24] Robin X. *et al.* pROC: an open-source package for R and S+ to analyze and compare ROC curves. BMC Bioinformatics, 12, 77 (2011). 21414208 10.1186/1471-2105-12-77 PMC3068975

