# Supplementary material for: Increased N-glycosylation of Asn88 in serum pancreatic ribonuclease 1 is a novel diagnostic marker for pancreatic cancer
Source: Sci Rep. 2014 Oct 22;4:6715. doi: 10.1038/srep06715 (PMC4205882; doi:10.1038/srep06715)
Supplement: Supplementary Information — Supplementary Figures [file srep06715-s1.pdf]

Increased *N*-glycosylation of Asn^{88} in serum pancreatic ribonuclease 1 is a novel diagnostic marker for pancreatic cancer

Daisuke Nakata[1]

AIA Research Group, Department of Reagent Development, Division of Bioscience, Tosoh Corporation

Supplementary Figure 1

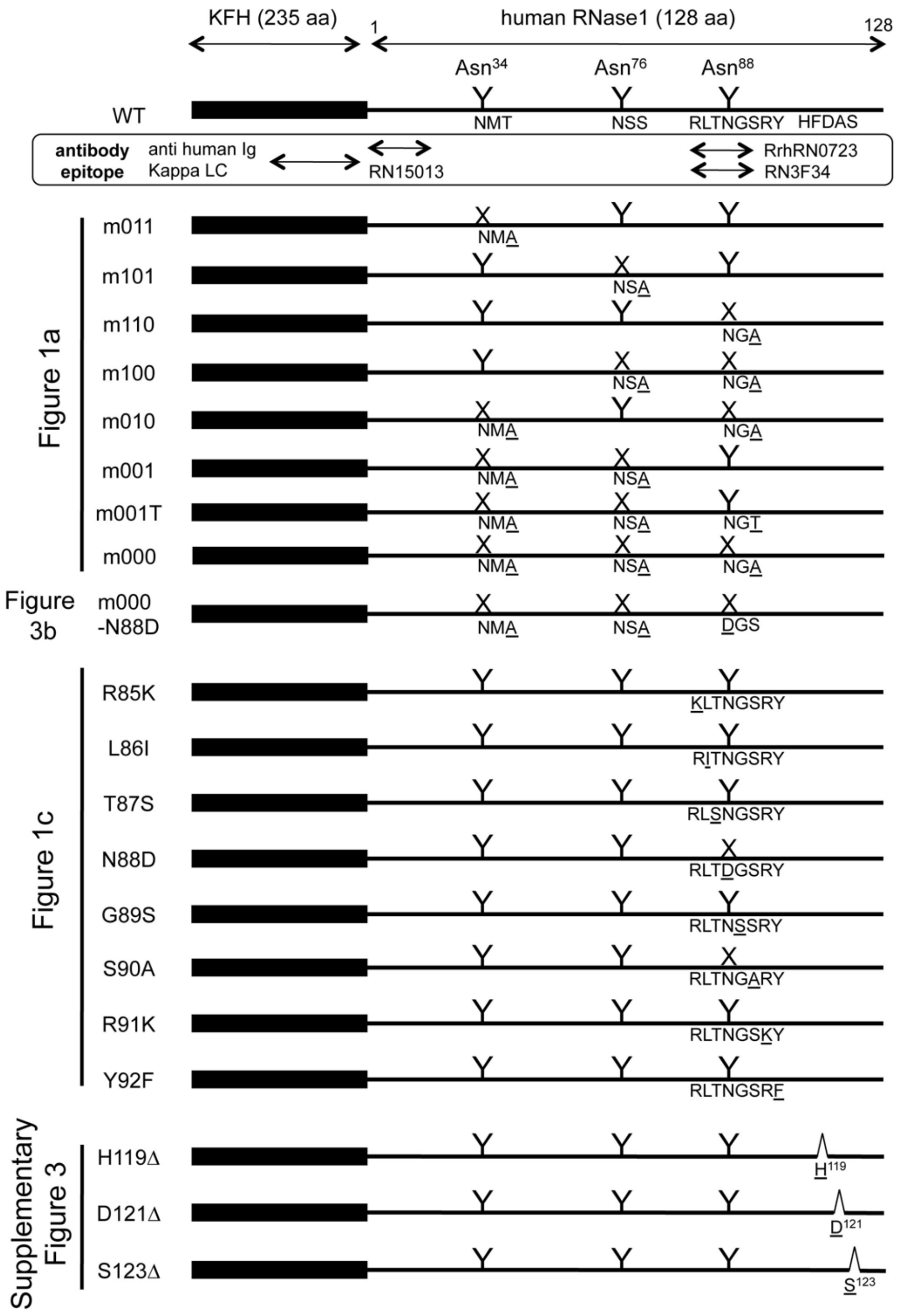

The illustrated diagrams for recombinant protein of wild-type and mutants of RNase1.
The illustrated diagram of wild-type RNase1 with *N*-terminal tags (human immunoglobulin kappa light chain linked to the FLAG and 6× His tags, KFH) are shown on top of figures. The epitopes for anti-human Ig Kappa LC, RN15013, RrhRN0723 and RN3F34 are also indicated, respectively. The mutated recombinant proteins used in this report are listed with their names. The substituted and deleted amino acids were indicated by the underlined letter in each diagram. Y indicates the intact *N*-glycosylation site. X indicates the defective *N*-glycosylation site by mutation.

Supplementary Figure 2

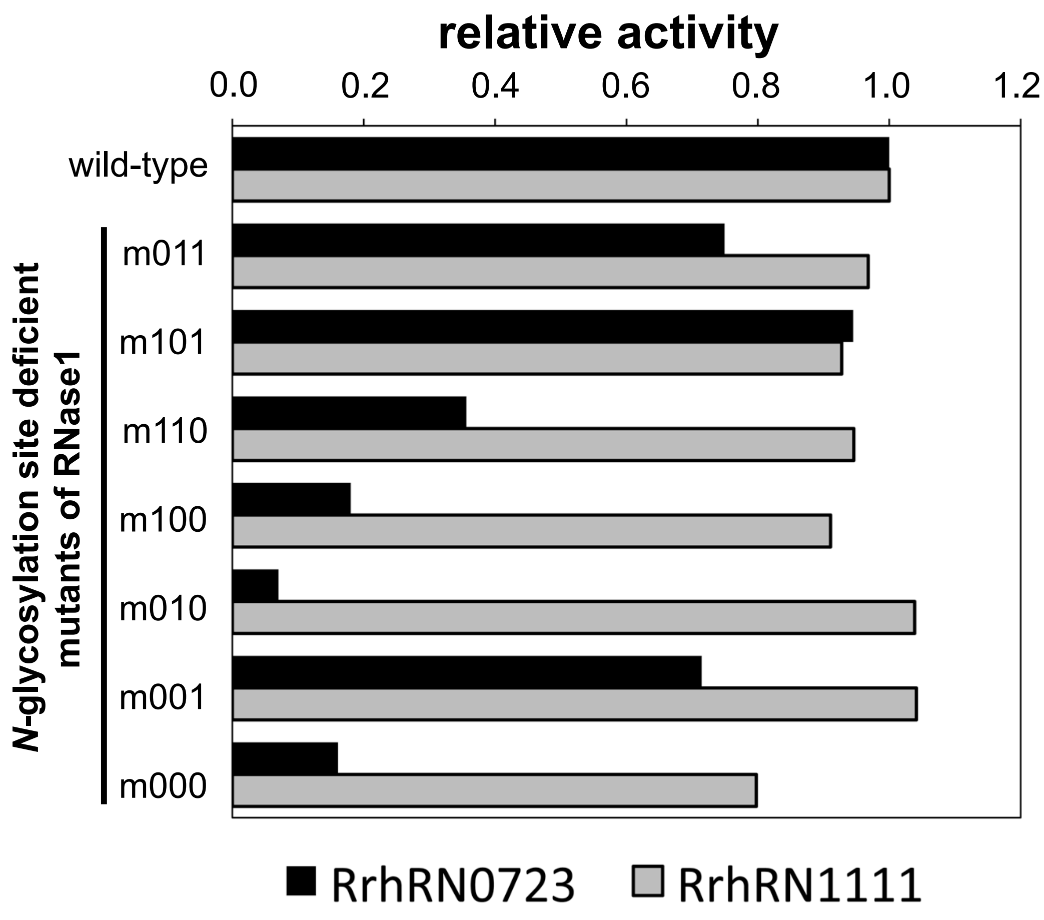

The reactivity of antibody RrhRN1111 with *N*-glycosylation-site deficient RNase1 mutants. The reactivities of antibody RrhRN1111 with seven *N*-glycosylation site-deficient mutants and wild-type proteins were analyzed using the ELISA described in Methods. The RrhRN0723 antibody was also used as a control. The values of antibody activities with the mutants are relative to that of the wild-type. The RrhRN1111 antibody binds RNase1 independent of its state of *N*-glycosylation.

Supplementary Figure 3

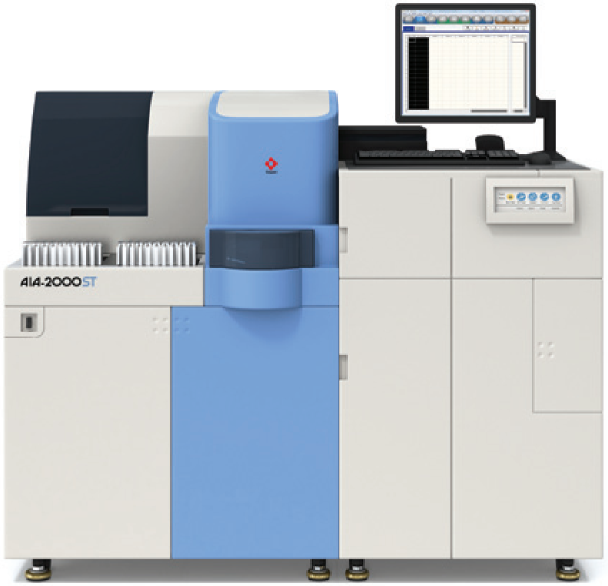

AIA-2000

The AIA system and test cup

The Automated Immunoassay Analyzer 2000 (AIA-2000) and AIA test cup are shown in left panel. AIA-2000 processes 200 tests one hour and is used worldwide in clinical laboratories. The AIA test cups shown below the instrument are used for the commercial CEA assay. A diagram of the test cup is shown in right-bottom panel. In the present study, 12 magnetic beads with immobilized antibody (MrhRN0614) and enzyme-labeled antibodies (RrhRN0723 or RrhRN1111) in buffer were freeze-dried in the cup and sealed with aluminum for use in the AIA. Use of all images in this figure were permitted by Bioscience Devision of Tosoh corporation.

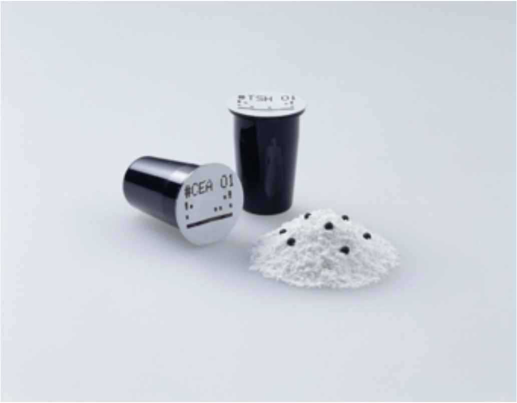

Test cup for AIA system
(commercial CEA test cup)

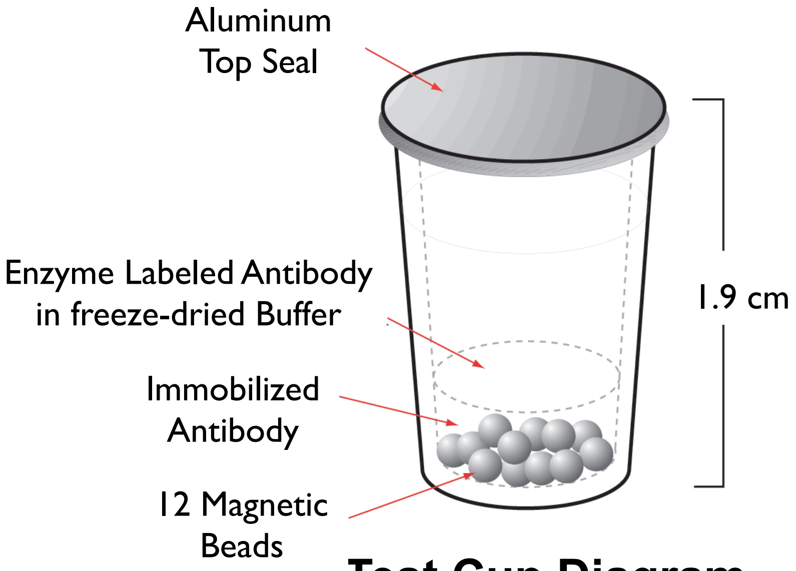

Test Cup Diagram

Supplementary Figure 4

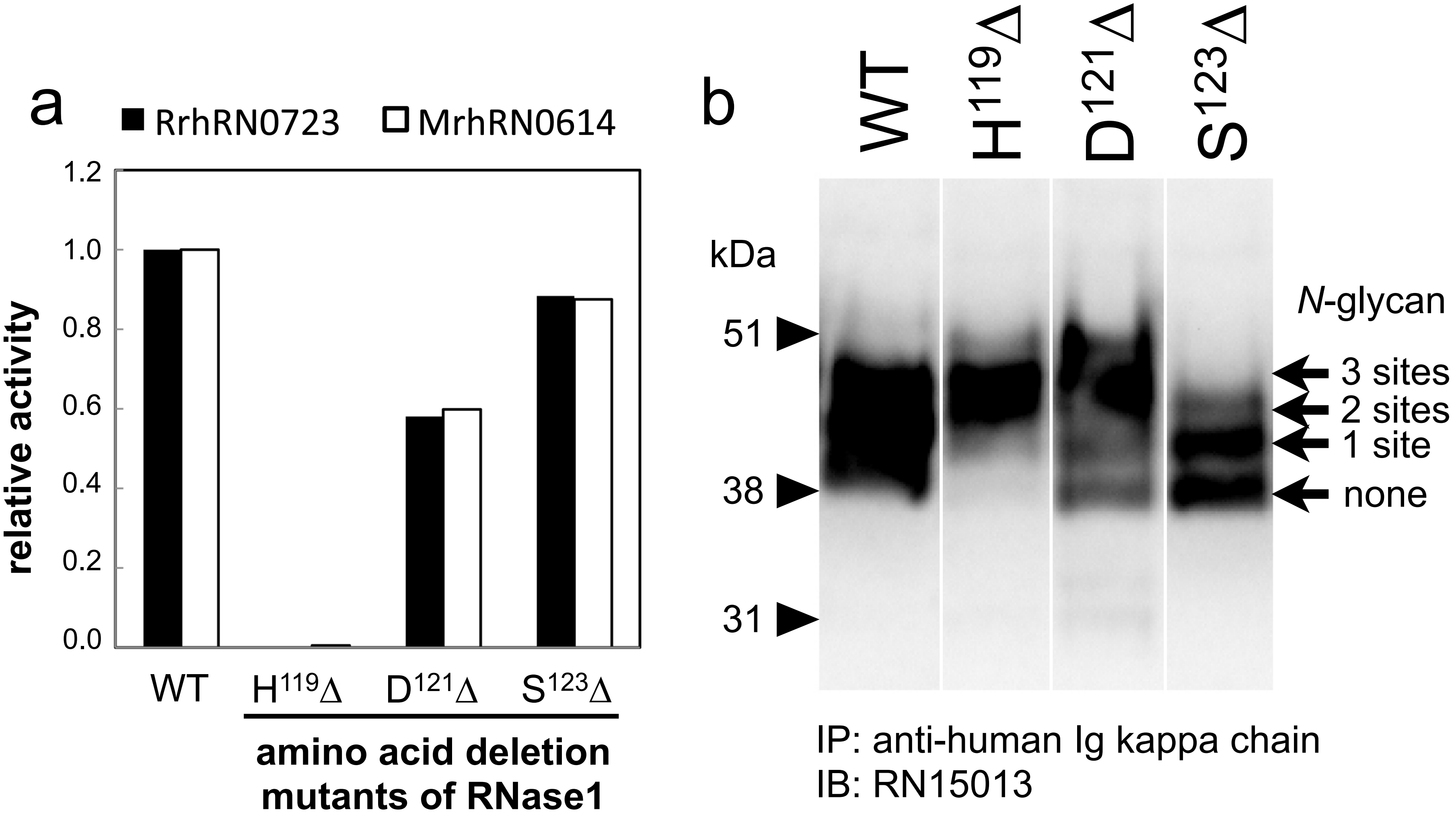

ELISA and western blot analyses of the RNase1 single amino acid-deletion mutants. The wild-type and deletion mutants ($H^{119}\Delta$, $D^{121}\Delta$ and $S^{123}\Delta$) were expressed in CHO-K1 cells and analyzed using ELISA and western blotting techniques described in Methods. The H^{119} Δ mutant was extensively *N*-glycosylated (panel *b*) and was not detected by either RrhRN0723 or MrhRn0614 antibody (panel *a*). The result of panel *b* was shown as a cropped image from the same blot membrane.

Supplementary Figure 5

Calibration data and curves for analysis of Asn[88]-free and total RNase1. The concentrations of the RNase1 standard isolated from CHO-K1 cells were determined using authentic Asn[88]-free and total RNase1 (master-control). Every lot of test cups was calibrated. The calibration curves were fit to a log-log cubic equation.

Asn[88]-free RNase1						total RNase1					
concentration (ng/ml)	rate (nM/sec)[1]			mean	SD (%)	concentration (ng/ml)	rate (nM/sec)[1]			mean	SD (%)
0.0	0.046	0.057	0.049	0.051	11.223	0.0	0.056	0.059	0.067	0.061	9.373
3.1	2.989	2.806	2.889	2.895	3.166	3.3	5.889	5.907	5.850	5.882	0.495
6.3	7.666	7.487	7.555	7.569	1.194	6.7	13.798	13.716	13.371	13.628	1.663
12.5	20.104	20.033	19.782	19.973	0.847	13.3	30.595	30.301	29.314	30.070	2.232
25.0	46.884	46.33	45.33	46.181	1.705	26.6	65.069	63.414	63.119	63.867	1.646
50.0	98.84	97.12	99.586	98.515	1.284	53.2	138.207	136.597	134.585	136.463	1.330
100.0	168.331	169.756	173.9	170.662	1.695	106.5	233.030	233.684	229.669	232.128	0.928

1: rate is the velocity of 4MU production by alkaline phosphatese/sec.

Calibration curve for Asn[88]-free RNase1

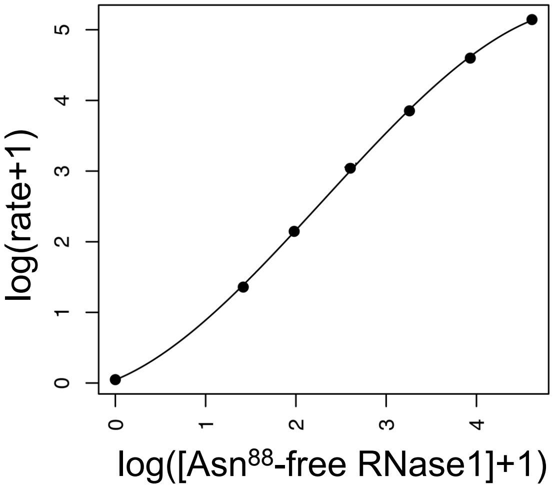

calibration curve

X = log([Asn[88] free RNase1]+1)
Y = log(rate +1)
$Y = A + BX + CX^2 + DX^3$
A = 0.04446
B = 0.52103
C = 0.37939
D = -0.05490

$R^2 = 0.99983$

Calibration curve for total RNase1

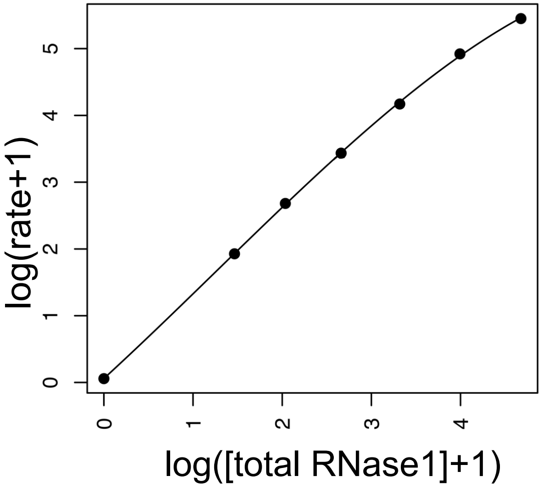

calibration curve

X = log([total RNase1]+1)
Y = log(rate +1)
$Y = A + BX + CX^2 + DX^3$
A = 0.05971
B = 1.22290
C = 0.06151
D = -0.01625

$R^2 = 0.99988$
